# Is the Profession of Dental Surgery in Its Relations to Young Men Who Are Entering Its Ranks Holding Its Own with Other Professions and Pursuits?

**Published:** 1875-09

**Authors:** Edgar Palmer

**Affiliations:** La Crosse


### IS THE PROFESSION OF DENTAL SURGERY IN ITS RELATIONS TO YOUNG MEN WHO ARE ENTERING ITS RANKS HOLDING ITS OWN WITH OTHER PROFESSIONS AND PURSUITS?

*Essay read before the Wisconsin State Dental Society. July 21, 1875.*

BY EDGAR PALMER, LA CROSSE.

This question forced itself upon my mind in the spring of 1873, while among the students of the Dental and Medical Colleges of Philadelphia, and has since clamored loudly for an answer. If it be a fact as it seemed apparent to me then, that a majority of the more studious and talented young men were rejecting the science and art of dental surgery, and that it was passing out of the hands of eminent and useful men whose labors have been and are so productive of rich treasures of thought and sentiment and practical knowledge, into the keeping of young men of less intellectual energy and ambition, what causes may be assigned as having a tendency to bring such a result, and what shall be the remedy?

So long as the world is upheld by the veracity of great and good men so long will the visions of youth and the occupation of manhood be directed in search of them, to get a glimpse of their works and if possible to waylay and entrap the secret of their magnetic influence and power. In every profession or pursuit, the rank and file take their cue and derive their stimulus from men of vigorous minds commanding presence, or imputed merit. We attach rank to the leaders of our profession in proportion to the quality of the ideas they embody in their productions or the genius they display in developing the hidden and expectant things of our science and art.

Emerson says: "I count him a great man who inhabits a higher sphere of thought into which other men rise with labor and difficulty."

We have great men in our profession to-day—men of culture,—that professional culture so beautifully represented as "the blossoming pathway leading from mind to progress, whose flowers are planted by discipline, weeded by disappointment and opened by the sunlight of Divine Love."

But we see these men few by few drop off from the stage of active life, and who shall fill their places—what is the character of those just setting out in the profession and how many are trying to fill the places of these eminent men, and succeed them in all their usefulness and honor?

I need not enter into an argument to prove that out of the many students found in our offices and attending the dental colleges at the present time but a very small number can be found who have taken up this profession because they were convinced the field presented to them an opportunity for intellectual development and scientific research, not to be found in other professions or pursuits, or where their talents with equal training and scope for exercise would not be more amply rewarded and appreciated.

No careful observer of educational tendencies can fail to see that the craving for honor, the title of dignity and respect, and the power and emoluments, which talent and professional refinement give are not sought after in our profession at the present time by young men of intellect and ambition to the extent that is seen in other professions and pursuits. And why?

Have young men learned to look upon the practice of dentistry as a kind of pursuit, which tasks only the bodily powers—a life in which the labor is only a physical toil—where the hands alone need training and the brain left in the background to rest in ignoble disuse?

Or is it because the practice of dentistry does not mean riches and thus fails to supply the stimulus to those who would combine wealth with honor and power, in the selection of a profession.

The young men who stand upon a pinnacle of ambition and progress, and look down upon the army of "Rubber-boilers" and charlatans who infest nearly every town, city, or hamlet in the country; who are not only poverty stricken themselves but by their impudence and peculiar style of solicitation manage to sap the life out of those who honor their calling, will find little to encourage them to adopt this profession as a means of wealth. These mountebanks say they have alienated from our profession that enterprise, wealth and honor we desire. We see in it no chance to rise and grow and be honored and appreciated; lower industries in a monetary point, offer better promises of wealth and with much less outlay and interval between the expenditure and the return.

And certain it is that the lust for gold can not be the ruling passion in the hearts of those who enter our ranks. But who shall say that the wealth and dignity of the dental profession can be measured by the money standard; or that there is not in it an equal opportunity for active minds to amass and achieve? Who among us can afford to see this profession pass into decadence?

When the profession of Dental Surgery shall be considered as inferior to the medical, or to other arts and sciences we have allowed a distinction, both disastrous and humiliating, and when civic honors, wealth and culture have been divorced from it the profession itself will have passed into lamentable debasement.

Let our educators and leaders show to the young men that notwithstanding the rags and filth which cling to us and the odium which these pretenders cast upon us there is here a market for the noblest powers and productions of man. That as brilliant prizes glitter in the sunlight of ambition in our profession as in any other.

The whole atmosphere of dental life is warm with provocations to think.

The last 20 years have been to us as to other arts and sciences, years of intense mental application. Like the chariot of Jove in ancient story "the flame that illuminated the path of its progress has been generated by the revolutions of our wheels." The inventions of our science are a marvel of the age and the opposition to nature's laws is so great that nothing less than incessant application will clear the way for to-morrow. Let it be shown also that it is a pursuit which can administer, as well to the higher and diviner side of man's nature. It is a school in that great University where the human faculties are in constant training; a school to increase knowledge that the heart may be enlarged,—to widen the field of investigation, to quicken thought, to substitute a coarse with a finer culture, and to yield, if rightly and faithfully followed, a wealth sufficient to satisfy just and honorable minds.

There is that in our daily practice which makes it dearer to us than the coin it brings. Our labors are full of little compensations which go far toward remunerating us for its cares perplexities and trials. With due attention to the laws of nature we enjoy health and peace of mind. The absence of tumult and temptation, the enjoyment of evening pleasures and home comforts and opportunities for the love and praise of Him who "directs sanctifies and governs all worthy undertakings."

Our profession, like every other calling, is what we choose to make it.

I believe it is in the power of every educated practitioner to mould and fashion the life of some one or more young men who shall rise to eminence, and usefulness, and compete for the honors which talent brings.

And just here I consider to be the true analysis of the question.

We are too apt to receive into our offices those young men whose most promising genius we can turn into dollars and cents, while they are serving their studentship.

Is it not the duty of every dentist who has been prospered and honored in his professional life to encourage and assist some worthy young man in the cultivation of mind and heart, and in a manner that shall lead him year by year into the highest exercise of his talent, and where he can find enduring wealth and pleasure.

Has not the world also a sacred claim upon us in this repect, for what is art, for what is culture, and for what purpose is all the genius and talent of our nations, if not to benefit the many and to disseminate the fruits which they alone can supply?

